# Evolution of determinant factors of maximal sprinting and repeated sprint ability in women soccer players

**DOI:** 10.1038/s41598-022-13241-x

**Published:** 2022-06-23

**Authors:** Francisco Tomás González-Fernández, Olalla García-Taibo, Moisés Vila, Hadi Nobari, Filipe Manuel Clemente

**Affiliations:** 1 Department of Physical Education and Sport, Faculty of Education and Sport Sciences, Campus of Melilla, University of Granada, 52006 Melilla, Spain; 2 Department of Physical Activity and Sport Sciences, Pontifical University of Comillas, CESAG, 07013 Palma, Spain; 3 Sports Scientist, Sepahan Football Club, Isfahan, 81887-78473 Iran; 4 Department of Exercise Physiology, Faculty of Educational Sciences and Psychology, University of Mohaghegh Ardabili, Ardabil, 56199-11367 Iran; 5 Faculty of Sport Science, University of Extremadura, Cáceres, Spain; 6 Department of Motor Performance, Faculty of Physical Education and Mountain Sports, Transilvania University of Braşov, 500068 Braşov, Romania; 7 Escola Superior Desporto e Lazer, Instituto Politécnico de Viana do Castelo, Rua Escola Industrial e Comercial de Nun’Álvares, 4900-347 Viana do Castelo, Portugal; 8 Instituto de Telecomunicações, Delegação da Covilhã, 1049-001 Lisboa, Portugal

**Keywords:** Physiology, Medical research, Engineering

## Abstract

The present study aimed to determine the influence of force–power–velocity, vertical and horizontal jumps, and repeated sprint ability on the sprinting performance of adult women soccer players. Eighteen women soccer players from one team participating in the first female national Spanish soccer league were analyzed. Fitness assessments were performed twice in a period of three months. The following assessments were made to reach the aim of the study: (1) anthropometric measures, (2) CMJ (0%, 20% and 40%), (3) hop test (dominant and nondominant leg), (4) linear sprinting at 30 m and (5) RSA test. The main evidence of this study revealed the meaningful contribution of lower-limb power (vertical and horizontal jump), maximal sprint and peak power on sprinting time performance, while stride frequency was meaningfully explained by vertical jump and maximal sprinting. In fact, positive moderate and large correlations were found between Time and CMJ, CMJ 20%, CMJ 40%, Hop Test Dominant and Non-dominant, and P_max_ and MS of Force–Power–Velocity (r = − 0.73, *p* = 0.001; r = − 0.68, *p* = 0.002; r = − 0.51, *p* = 0.03; r = − 0.64, *p* = 0.004; r = − 0.57, *p* = 0.013; r = − 0.78, *p* = 0.001, and r = − 0.83, *p* = 0.001, respectively). In sum, peak power, maximal speed, and lower-limb power (in vertical and horizontal jumps) were significant determinants of sprinting performance (time), while vertical jump was the determinant of stride frequency. In addition, our findings suggest that potentiation and explosive vertical power could be the emphasis for sustaining the stride frequency of women soccer players, while sprinting performance should be supported by strong acceleration and maximal velocity sustained by both vertical and horizontal force and concentric and eccentric strength and power.

## Introduction

During soccer competition, the intermittent characteristic of games combines low-intensity activity (e.g., walking, jogging at low-to-moderate intensity) with high-intensity actions (e.g., sprinting, jumping, accelerations and decelerations)^1–4^ that normally are determinant in games^5^. Accordingly, the capacity to perform maximal sprinting and repeated sprint ability in women soccer players are key to highlighting the individual capacity of each player^6,7^. Both high acceleration and maximal sprint velocity are two important components of sprint performance that could determine success in a decisive situation in soccer and enable winning the ball from opponents^8^. These capacities are a topic of great interest to coaches to properly structure and control the training load. Repeated-sprint ability (RSA) is the capacity to repeatedly produce maximal or submaximal sprints spaced over time with short recoveries during a game^9^. Consequently, women soccer players’ ability to withstand repeated maximal sprint efforts will be essential to provide better athletic performance^6,10^. A review of the literature reveals that the performance of RSA has a strong correlation with sprinting skills and high-intensity performance^11^. For this reason, RSA and linear sprint characteristics cannot be separated to investigate which factor is more determinant.

As we previously commented, to sustain the requirements and demands of a match, female soccer players should show a developed fitness status. A review of the literature reveals that female soccer players must possess high values of maximal oxygen uptake to be able to maintain all high-intensity efforts during the match^12^. In this respect, it should be noted that high-intensity efforts, normally shown in maximal sprints, are performed during a critical moment of the competition^13^. Indeed, the direction and magnitude of high-intensity effort seem to focus on the quantity and quality of sprints during the match^14,15^ and the quantity of meters covered^16^. In addition to sprinting capacity, jumping and hopping power are determinants of soccer performance. In fact, jump power is a greater predictor of sprint ability^17^. Predictions between jump ability and sprinting performance have been investigated and, in this regard, the relationships between hop (horizontal) and jump (vertical), maximizing running speed performance, stride length (SL), stride frequency (SF), and the kinematics of sprinting, have received a great deal of attention in the scientific literature over the years^18^.

Athletic performances, such as sprint performances, are complex tasks to evaluate involving a multitude of mechanisms, and consequently, the literature cites several studies focusing on this topic. Regarding vertical jumps, the relative force variable (peak force) obtained in countermovement jumps was presented to be a predictor for maximal running velocity through V_max_ 10-m and 60-m time^19^. Furthermore, elite sprinters have been shown to produce higher peak velocities and jump heights in comparison with significantly stronger elite powerlifters when performing countermovement jumps with external loads^20^. Thus, identifying whether strength and power outputs in gym-based exercises, such as diverse types of jumps, are related to sprinting ability has become of particular interest to many coaches and researchers in the area of sport sciences. This may provide greater insight into those exercises or variables that offer a superior training stimulus in terms of transference of gym-based gains to improving sprint ability^21^.

Great sprinting outcomes require a successful starting ability, high maximum velocity, and the capacity to maintain that velocity over time. Maximum muscle contraction force provides the mechanical power needed for the starting speed and short sprints^22^. This affirmation suggests that considering the stretch–shortening cycle is a key factor in developing acceleration during running. This could be explained by the neuromuscular improvement obtained in jump training and transferred into sprinting performance. Transferring the greatest explosiveness to sprinting is one of the most important goals of coaches with their athletes^23^. With respect to hops or horizontal jumps, it has been observed that this kind of task is related to the starting acceleration of the sprint, which should be considered in terms of the training process^24^. Thus, horizontal jump exercises have also been suggested to be incorporated into training routines to improve sprint performance^18^.

In sum, many investigators have taken this into consideration, resulting in a rapid growth of interest in the relations between RSA and maximal sprinting in women soccer players. However, to the best of our knowledge, very few studies have investigated the influence of force–power–velocity, vertical and horizontal jumps and repeated sprint ability on the sprinting performance of adult women soccer players. Knowing the relationships between jumping/hopping performance and sprinting variables could reveal which indicators are more relevant for coaches to refine their training prescription and identify which jump and hop exercises should be incorporated into a daily routine to improve sprint performance^18^.

On the basis of previous research, the purposes of this study were (1) to investigate the influence of force–power–velocity, vertical and horizontal jumps and repeated sprint ability on the sprinting performance of adult women soccer players, (2) to understand the relationship between sprinting variables and force–power–velocity, CMJ, hop test and RSA test, and (3) to run a regression analysis to explain which fitness variables could be used to better explain the importance of different sprinting variables. In this regard, the hypothesis of this work is that maximal sprinting variables may determine both jump capacity (horizontal and vertical) and RSA ability.

## Methods

### Study design and experimental approach

An observational analytic cohort design was used in the present study. Fitness assessments were performed twice during the intervention, with 3 months between the first and second assessments (September–December). The aim was to explore the variations (pre-post) of (1) CMJ (0%, 20%, 40%), (2) hop test, (3) linear sprinting (time, stride frequency, stride distance), (4) force–power–velocity (Peak power maximal (P_max_) and maximal speed, and (5) RSA [maximal Power (P_max_); minimum power (P_min_), and fatigue index (FI)]. All the participants completed all the assessments.

### Participants

Eighteen women soccer players belonging to the same team participating in the second division of the Spanish league agreed to participate in the study. The following inclusion criteria were applied to select the final sample: (1) a background of ≥ 5 years of systematic soccer training and competitive experience, (2) continuous soccer training for the previous 3 months and players who had had injuries or illness no longer than 4 consecutive weeks, (3) absence of potential medical problems, and (4) participants were required to attend ≥ 85% of all training sessions and attend all assessment sessions. Concerning the sample, mentions must be made that it included six defenders, six midfielders, and five attackers. Before starting the season, participants presented a mean age of 21.00 ± 4.18 years old and height of 165.55 ± 6.70 cm. In the first assessment, the mean weight was 61.33 ± 8.55 kg, and the body mass index was 22.29 ± 2.08 kg. In the second assessment, the mean weight was 62.22 ± 8.82 kg, and the mean body mass was 22.62 ± 2.33 kg. Lastly, these players trained twice a week (90 min per session) and played one official match a week. Generally, training sessions comprised a warm-up, main part, and cool down.

All the players were informed about the main aims of the investigation and signed informed consent forms. The students were treated according to American Psychological Association (APA) guidelines, which ensured the anonymity of participants’ responses. In addition, the study was conducted in accordance with the ethical principles of the Helsinki Declaration for Human Research and was approved by a scientific council of the local university (code: 2021/65).

### Data collection

**Figure 1:**
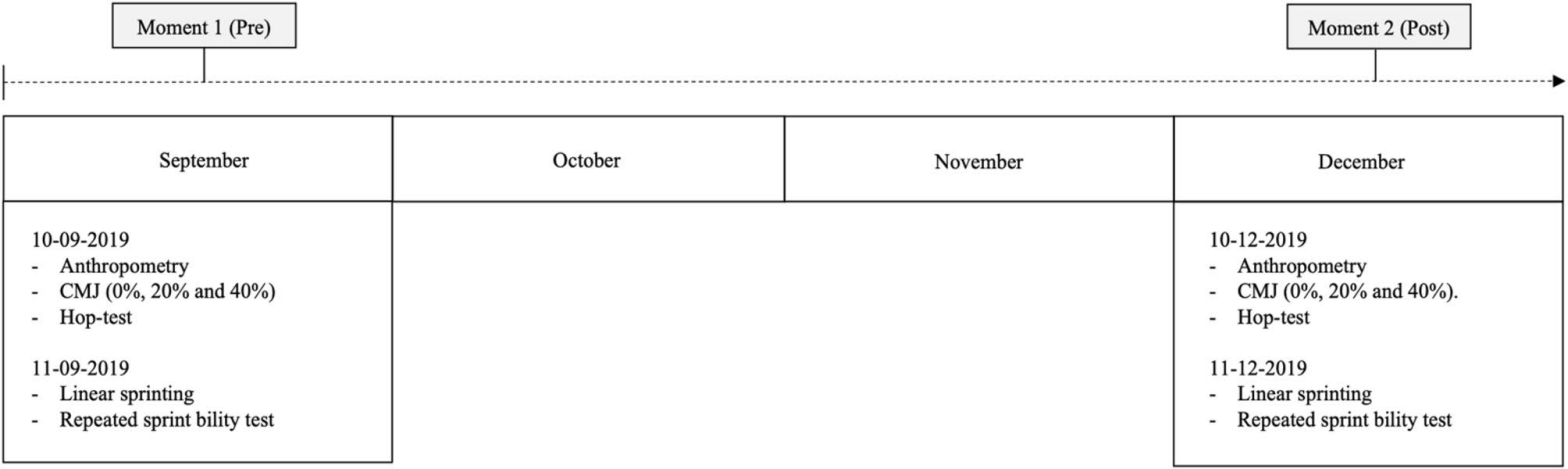
Timeline of the study.

The tests were recorded for two assessments, always at the same time and days of the week (7:30–9:30 p.m.). Consequently, the last match was considered for giving a rest period of 48 h to the women soccer players. In addition, both assessments were controlled and preceded by the same type of microcycle to avoid fatigue effects. Each assessment was divided into two moments: (1) Anthropometric measurement, CMJ (0%, 20%, 40%) and hop tests were evaluated in the first training session of the week, and (2) linear sprinting and repeated sprint ability tests were evaluated in the other training session of the week. Anthropometric measurement, countermovement jumps and hop tests were performed in a private room with a stable temperature of 22 °C and relative humidity of 52%. The 30 m linear sprinting and repeated sprint ability tests were performed on a synthetic turf field with a mean temperature of 17.7 ± 3.1 °C and relative humidity of 71 ± 3%. No windy or rainy conditions were experienced in the assessment. To record data, we followed the protocol established by Gonçalves et al.^6^ for anthropometric measurement, countermovement jumps and linear sprinting. However, we implemented two tests as hop tests and repeated sprint ability tests proposed by Bangsbo with a change-of-direction test (See Fig. 1, for more information).

### Anthropometric measurement

Body weight (kg) was measured without shoes with a bioelectrical impedance analysis (BIA) device (Tanita BC-730) to the nearest 0.1 kg. Height (cm) was measured using a stadiometer (Type SECA 225, Hamburg, Germany) to the nearest 0.1 cm. Body mass index was calculated as body weight (in kg) divided by height squared (in m).

### Countermovement jump

The CMJ was evaluated as follows. After a warm-up, the women soccer players performed the CMJ at different weights: 0% of body weight (with hands holding a bar), 20% of body weight (hands holding a weighted barbell), and 40% of body weight (hands holding a weighted barbell). The players performed three jumps with every load with 30 s of recovery between attempts and 3 min between the different load jumps. In addition, the jump order was counterbalanced to avoid order effects. Note that participants were instructed to jump as high as possible after reaching a knee angle of ~ 90°. Participants were also instructed to keep the position mentioned above during the CMJ and to land with their legs extended with maximal foot plantar flexion. If any of these requirements were not met, the trial was repeated. To minimize the effect of fatigue, 30 s of recovery was provided between consecutive trials. The best jump (in cm) was considered the final outcome. Chronojump-Boscosystem^®^ (Barcelona, Spain) developed by De Blas et al.^25^ was used to evaluate the CMJ. This system was connected to a MacBook Pro (macOS Sur 11.1). In addition, the measures were analyzed by chronopic and recorded by Chronojump version 2.0.2.

### Hop test

According to previous suggestions by Rösch et al.^26^, athletes performed Hop D and Hop non-D with arm swing. Athletes performed three single maximal horizontal jumps with the dominant and non-dominant legs landing on one leg. Three maximal valid attempts were allowed, with 3 min rest in between. Performance was measured using a measuring tape secured to the floor. The best performance was retained for further analysis.

### Linear sprinting

The 30-m sprints were evaluated using the *MySprin*t app^27^. To ensure successful performance, we followed the protocol of Samozino et al.^27^. The aim of this test was to run 30 m as fast as possible. A previous study revealed that no significant difference is revealed in women soccer players sprint time between 20–30 and 30–40 m^11^. Additionally, soccer players tend to accelerate, while reaching peak speed sooner (between 15 and 21 m)^28,29^ than in other sports (such as track and field sprints). The women soccer players started from a crouching position with the right hand on the field, were instructed to sprint at maximum speed and were given two attempts for each condition. We recorded the best of the two attempts (measured in seconds by the *MySprint* app and Ipad Pro model A1673 (iOS 13.3.). A camera (HD of 1080 p 240 fps) was used to record and analyze all attempts.

### Repeated sprint ability test by Bangsbo with a change-of-direction test

The protocol used for testing RSA in the present study used a modified version of the protocol first introduced by Bangsbo^30^, which was performed on synthetic turf. The protocol used for testing the RSA consisted of 30 linear meters (with change-of-direction), performed seven times and with a recovery time between efforts of 10 s. The participants started their sprint 0.5 m behind the start timing gate. Microgate Wireless Training Timers (Microgate, Bolzano—Italy), with digital FSK transmission; redundant code with information correctness verification and autocorrection, multifrequency transceiver 433–434 MHz and impulse transmission accuracy ± 0.4 ms, were positioned at the beginning and end lines to record the time of each sprint. The time (s) for each trial was recorded. After that, minimum and maximum peak power were determined using the equation^31^. $\mathrm{Power }= \frac{\mathrm{Body mass }\times {\mathrm{Distance}}^{2}}{{\mathrm{Time }}^{3}}$, and the fatigue index used the following equation $\mathrm{Fatigue index}= \frac{{\mathrm{max}}_{\mathrm{power}}- {\mathrm{min}}_{\mathrm{power}} }{\mathrm{Sum of }6\mathrm{ sprints }(\mathrm{s})}$.

### Statistical procedures

The mean and standard deviation were used for data processing. Descriptive statistics were calculated for each variable (see Table 1 for more information). Before any parametric statistical analysis was performed, the assumption of normality was tested with the Kolmogorov–Smirnov test on each variable. In relation to the changes over the season, a paired sample *t*-test was used for determining differences as a repeated measures analysis (Moment 1–Moment 2). Cohen d was the effect size indicator. To interpret the magnitude of the effect size, we adopted the following criteria: d = 0.20, small; d = 0.50, medium; and d = 0.80, large. A Pearson correlation coefficient *r* was used to examine the relationship between RSA (P_max_, P_min_, and FI) and the remaining variables (CMJ, Hop test, 30 m sprint, RSA test). To interpret the magnitude of these correlations, we adopted the following criteria: *r* ≤ 0.1, trivial; 0.1 < *r* ≤ 0.3, small; 0.3 < *r* ≤ 0.5, moderate; 0.5 < *r* ≤ 0.7, large; 0.7 < *r* ≤ 0.9, very large; and *r* > 0.9, almost perfect. Confidence intervals (95% CI) were calculated for each correlation. Multiple regression analysis was used to model the prediction of RSA from the remaining variables. In this regression analysis, all variables were examined separately. Data were analyzed using Statistica software (version 13.3; Statsoft, Inc., Tulsa, OK, USA).

**Table 1:** Anthropometric measurement and fitness variables at the two moments of assessment (mean ± SD).

Women soccer players (n = 17)				
	Moment 1	Moment 2	UCI|CI|LCI (95%)	t-test|Cohen d
**Anthropometric measurement**				
Weight (kg)	61.33 ± 8.55	62.22 ± 8.82	65.82|4.05|57.72	*p* = 0.20|*d* = 0.09
Body mass index (%)	22.29 ± 2.08	22.62 ± 2.33	23.46|1.01|21.45	*p* = 0.31|*d* = − 0.15
**Countermovement jump**				
CMJ (cm)	23.72 ± 2.23	25.07 ± 3.20	22.98|1.20|24.19	*p* = 0.03*|*d* = − 0.49
CMJ 20% (cm)	17.62 ± 2.70	18.42 ± 2.11	18.90|1.10|16.70	*p* = 0.41|*d* = − 0.33
CMJ 40% (cm)	13.99 ± 2.83	14.69 ± 1.99	15.26|1.16|12.93	*p* = 0.57|*d* = − 0.29
**Hop test**				
Dominant (cm)	122.06 ± 7.35	136.42 ± 12.95	132.09|4.37|123.35	p = 0.001**|*d* = − 1.36
Non dominant (cm)	123.88 ± 8.99	140.85 ± 10.50	125.92|4.25|134.42	p = 0.001**|*d* = − 1.74
**Linear sprinting (30 m)**				
Time (s)	5.21 ± 0.18	5.18 ± 0.19	5.28|0.08|5.12	p = 0.44|*d* = 0.17
Stride frequency (n)	3.74 ± 0.21	3.64 ± 0.29	3.83|0.11|3.61	p = 0.15|*d* = 0.41
Stride length (m)	1.54 ± 0.09	1.59 ± 0.11	1.59|0.04|1.51	p = 0.10|*d* = − 0.54
Force–power–velocity				
P_max_ (W/kg)	13.37 ± 1.83	13.44 ± 1.85	14.04|0.82|12.40	p = 0.25|*d* = − 0.04
Maximal speed	6.96 ± 0.30	7.03 ± 0.27	7.11|0.12|6.87	p = 0.25|*d* = − 0.24
**RSA test**				
P_min_ (s)	138.49 ± 21.41	152.74 ± 18.91	152.81|10.59|131.64	p = 0.03*|*d* = − 0.71
P_max_ (s)	167.58 ± 22.79	186.53 ± 17.32	185.03|11.48|162.07	p = 0.001**|*d* = − 0.94
FI (%)	0.58 ± 0.21	0.70 ± 0.24	0.73|0.10|0.53	p = 0.03*|*d* = − 0.52

*UCI* upper confidence interval, *CI* confidence interval, *LCI* lower confidence interval, *CMJ* countermovement jump, *30 m* 30-m sprint, *force–power–velocity* peak power (P_max_) and maximal sprint, *RSA* repeated sprint ability, *P*_*min*_ peak power (minimum), *P*_*max*_ peak power (maximum), *FI* fatigue index.

*Significance at *p* < 0.05.

**Significance at *p* < 0.01.

### Ethics approval and consent to participate

The study was conducted according to the guidelines of the Declaration of Helsinki and approved by the Research Ethics Committee of the University of Comillas (code: 2021/65). After obtaining approval, we invited all the people responsible for the team and families to a meeting in which we presented the objectives of the project and asked them to sign an informed consent form. Parents, team staff and coaches were informed that they could revoke the participation agreement at any time. Every young soccer player was verbally informed and asked to provide consent prior to the completion of each test and intervention.

### Consent for publication

No individual or indemnifiable data are being published as part of this manuscript.

## Results

Descriptive statistics were calculated for each variable (see Table 1, for more information).

**Figure 2:**
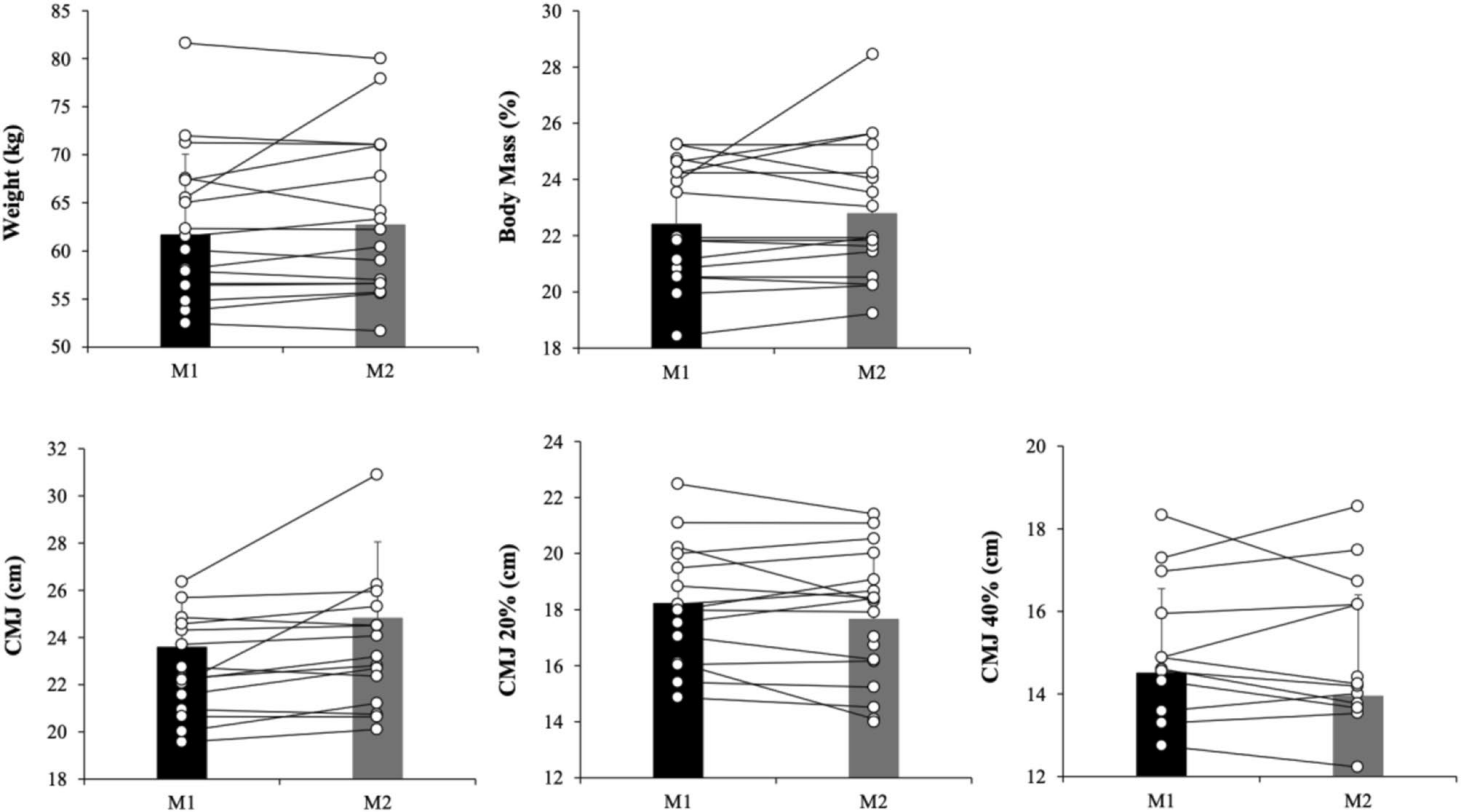
Performance variables (anthropometric measurements and countermovement jump) in moment 1 (M1) and Moment 2 (M2).

A paired measures *t-*test with participants’ mean anthropometric measures (1) weight and (2) body mass index did not reveal significant effects, *p* = 0.20, *d* = 0.09 and *p* = 0.31, *d* = − 0.15, respectively. A new paired measures *t-*test with participants’ mean countermovement jumps with different loads (1) CMJ, (2) CMJ 20% and (3) CMJ 40% revealed a significant effect on CMJ 40%, *p* = 0.03, *d* = − 0.49. However, the *t*-test for CMJ 20% and CMJ 40% did not reveal significant effects, *p* = 0.41, *d* = − 0.33 and *p* = 0.57, *d* = − 0.29, respectively (See Fig. 2).

**Figure 3:**
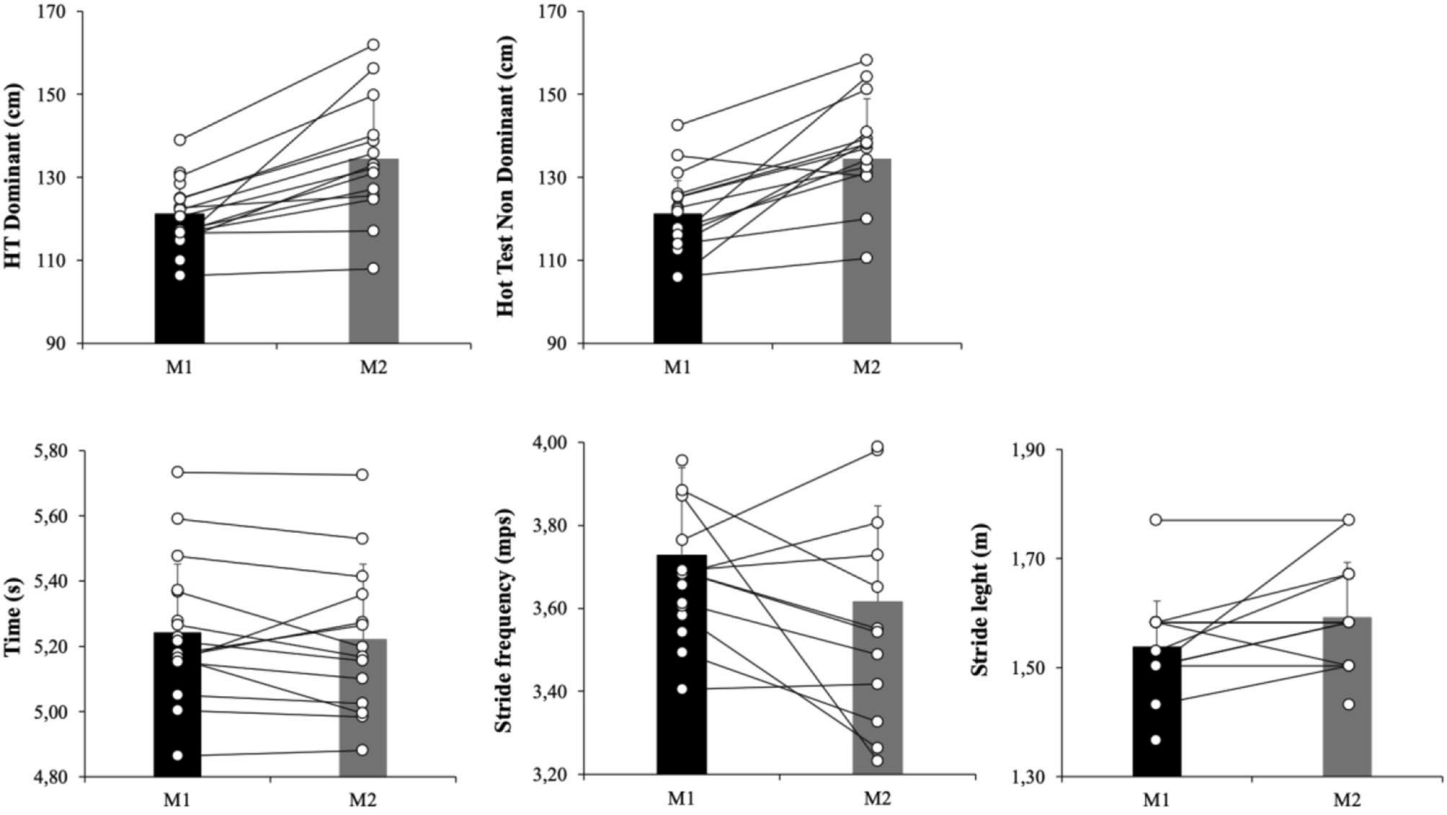
Performance variables (Hop test and Linear Sprinting 30-m) in moment 1 (M1) and Moment 2 (M2).

Crucially, another paired measures *t-*test with participants’ mean Hop test dominant and Hop test Non dominant did reveal significant effects, *p* = 0.001, *d* = − 1.36 and *p* = 0.001, *d* = − 1.74, respectively. The paired measures *t-*test with participants’ mean linear sprinting (1) time, (2) stride frequency and (3) stride length did not reveal significant effects, *p* = 0.44, *d* = 0.17, *p* = 0.15, *d* = − 0.41 and *p* = 0.10, *d* = − 0.54, respectively. Another paired measures *t-*test with participants’ mean force–power–velocity (1) P_max_ and (2) Maximal speed did not show significant effects, *d* = 0.25 and *p* = − 0.04, *d* = − 0.24, respectively. For more information, see Fig. 3.

**Figure 4:**
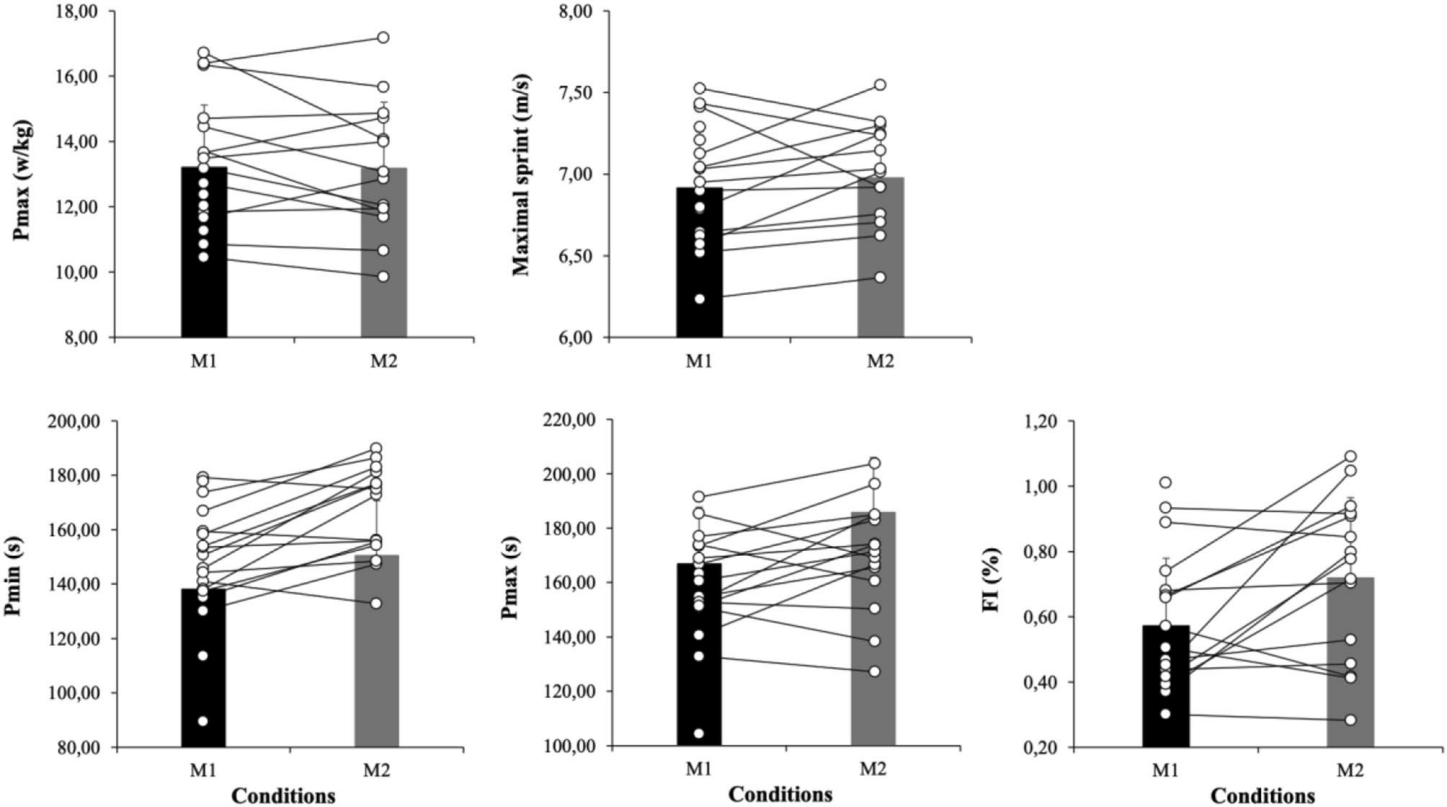
Performance variables (force–power–velocity and RSA test) in moment 1 (M1) and Moment 2 (M2).

Last, a new paired measures *t-*test with participants’ mean RSA test (1) P_min_, (2) P_max_, and (3) FI showed significant effects on P_min_, P_max_ and FI, *p* = 0.03, *d* = − 0.71, *p* = 0.001, *d* = − 0.94 and *p* = 0.03, *d* = − 0.52, respectively (See Fig. 4).

Sprinting variables (time, SF, and SL) and force–power–velocity, CMJ, hop test and RSA test are summarized in Table 2. On the one hand, no significant correlations were found between all Sprinting variables and RSA measures (P_min_, P_max_, and FI). In addition, no significant correlations were found between SF and dominant hop test and nondominant hop test. Moreover, no significant correlations were shown between SF and P_max_ of force-power velocity in SF and SL. However, negative very large correlations were found between Time and P_max_ and MS (r = − 0.74, *p* = 0.001; r = − 0.75, *p* = 0.001). Crucially, positive large correlations were found between SF and CMJ, CMJ 20%, and CMJ 40% (r = 0.62, *p* = 0.01; r = 0.60, *p* = 0.01 and r = 0.67, *p* = 0.003, respectively). Finally, no significant correlations were found between SL and CMJ, CMJ 20%, hop test dominant and nondominant, and P_max_ and MS of force power velocity. Although, negative moderate correlations were found between SL and -CMJ 40% (r = − 0.48, *p* = 0.04).

**Table 2:** Pearson correlation coefficient between sprinting variables and force–power–velocity, CMJ, hop test and RSA test (n = 17).

	Force–power–velocity		CMJ			Hop test		RSA test		
	P_max_	MS	0%	20%	40%	D	ND	P_min_	P_max_	FI
Time	r = − 0.74 p = 0.001**	r = − 0.75 p = 0.001**	r = − 0.74 p = 0.001**	r = − 0.69 p = 0.002**	r = − 0.45 p = 0.63	r = − 0.51 p = 0.03*	r = − 0.40 p = 0.10	r = − 0.12 p = 0.63	r = 0.14 p = 0.58	r = − 0.15 p = 0.56
SF	r = 0.15 p = 0.55	r = 0.42 p = 0.09	r = 0.62 p = 0.01*	r = 0.60 p = 0.01*	r = 0.67 p = 0.002**	r = 0.16 p = 0.51	r = 0.23 p = 0.35	r = − 0.24 p = 0.36	r = 0.17 p = 0.49	r = 0.12 p = 0.62
SL	r = 0.55 p = 0.20	r = 0.09 p = 0.06	r = − 0.21 p = 0.40	r = − 0.26 p = 0.31	r = − 0.48 p = 0.04*	r = 0.07 p = 0.78	r = − 0.09 p = 0.72	r = 0.37 p = 0.13	r = 0.32 p = 0.20	r = − 0.03 p = 0.88

*CMJ* countermovement jump, *force–power–velocity* peak power (P_max_) and maximal sprint (MS), Hop Test D (dominant) and Hop Test ND (Non-Dominant), *RSA* repeated sprint ability, *P*_*min*_ peak power (minimum), *P*_*max*_ peak power (maximum), *FI* fatigue index.

*Significance at *p* < 0.05.

**Significance at *p* < 0.01.

Finally, a multiple regression analysis (Table 3) was performed to verify which fitness variables (agreement with the correlation analysis), could be used to better explain the importance of different sprinting variables (Time, SF, and SL). On the one hand, multiple regression for Time revealed significant effects for CMJ, CMJ 20%, HT D, P_max_ and MS (r = 0.74, r = 0.69, r = 0.51, r = 74 and r = 74, respectively. On the other hand, multiple regression for SF showed significant effects for CMJ, CMJ 20% and CMJ 40% (r = 0.62, r = 0.60 and r = 67, respectively). Last, another multiple regression for SL revealed significant effects for CMJ 40% (r = 0.48). For more information, see Table 3.

**Table 3:** Values of regression analysis explaining the relevance of different sprinting variables (time, SF, and SL).

	R	R^2^	Adjusted R^2^	F	P	SE
**Time**						
CMJ	0.74	0.56	0.53	19.27	0.001**	0.17
CMJ 20%	0.69	0.47	0.44	14.23	0.002**	0.19
HT D	0.51	0.26	0.21	5.39	0.03*	0.22
P_max_	0.74	0.52	0.52	18.56	0.001**	0.17
MS	0.74	0.55	0.52	18.80	0.001**	0.17
**SF**						
CMJ	0.62	0.38	0.34	9.51	0.01*	0.20
CMJ 20%	0.60	0.36	0.32	8.59	0.01*	0.20
CMJ 40%	0.67	0.45	0.42	12.71	0.002**	0.18
**SL**						
CMJ 40%	0.48	0.23	0.18	4.61	0.04*	0.22

*Significance at *p* < 0.05. **Significance at *p* < 0.01.

## Discussion

This study aimed to determine the influence of force–power–velocity, vertical and horizontal jumps and repeated sprint ability on the sprinting performance of adult women soccer players. The main evidence of this study revealed the meaningful contribution of lower-limb power (vertical and horizontal jump), maximal sprint and peak power on sprinting time performance, while stride frequency was meaningfully explained by vertical jump and maximal sprinting. Research about determinants of sprinting performance in soccer, is mostly conducted on men^32^ while few have centered on women^33^.

The ability to perform sprints and highly demanding actions in soccer is well known, namely, to support crucial events of the match such as counterattacks or fast transitions^34^. Additionally, a progressive increase in the volume of actions made in high-intensity running, in which sprinting is included, has been observed^35^. Therefore, players have become faster to support the demands of the match and to compete at the highest levels^36^, mainly considering that important situations such as goals are preceded by sprinting^5^. Therefore, knowing the determinants of sprinting, it is important to identify which physical qualities should be emphasized to support this capacity.

As an example, a previous study conducted on women soccer players suggested that unilateral jumping tests were more strongly correlated with sprinting performance than bilateral tests^33^. However, despite the associations between sprint time and physical qualities representing key information, further research about qualitative information on sprinting is needed. Thus, we have added other measures such as stride frequency and length which may explain better the relationships with some specific physical qualities.

The current study revealed that sprinting variables (time, stride frequency and length) were significantly correlated with different outcomes. As an example, sprinting time was very highly correlated with the maximal peak and maximal sprint in the force–power–velocity dimension, as well as with countermovement jumps with 0% and 20% load and hop tests. On the other hand, stride frequency was highly correlated with countermovement jumps with 0 and 20% loads and very highly correlated with countermovement jumps with 40% loads. The stride length was only moderately correlated with the countermovement jump at a 20% load. Such evidence is supported by previous findings which suggests that faster players reach higher absolute running speeds^37^, while the stretch–shortening cycle present in countermovement jumps can support sprinting performance^38^, namely in peak speed in which greater reactive strength is predominant^39^.

Sprinting performance (time) is an overall measure dependent on two main sprinting phases^40^: acceleration and maximal velocity. Usually, acceleration presents a longer ground contact time, shorter stride length, and frequencies^41^. While maximal speed is more dependent on vertical force production, influencing stride length and frequencies; acceleration is more dependent on horizontal forces in which an explosive concentric strength is required to overcome inertia^41^. During maximal velocity, more explosive muscular stiffness and elasticity is expected that supports the decrease in ground contact time and increase in ground reaction force^42^.

Such facts may explain why stride frequency was strongly correlated with countermovement jumps with no load or the smallest load (20%). Considering that stride frequency requires a more explosive and reactive strength, it is natural that potentiation can be a better contributor to the countermovement jump^18^. However, the same movement with a moderate load (40%) becomes more dependent on concentric force to elevate, thus reducing the contribution of potentiation and, for this reason, decreasing the association with stride frequency. Therefore, it is plausible to admit that stride frequency is more dependent on reactive strength and potentiation; and a countermovement jump without a heavy load may better contribute to the higher stride frequency performance.

Sprinting time is dependent on different factors, such as maximal speed or strong acceleration^24^. Both are relatively independent^21^, and the dependency on different physical qualities is justified^35^. Horizontal forces are determinant in acceleration^43^. This may explain the large correlations with the hop test, in which concentric horizontal forces are strongly present to overcome inertia. On the other hand, vertical forces become prevalent with the progression of sprinting^44,45^. This may explain the very large correlations of sprinting time with countermovement jump (mainly without load and with low load) and with maximal peak power and maximal speed that supports the capacity for moving faster.

This study had some limitations. The sprinting time was not split into phases; thus, acceleration was not analyzed separately from maximal velocity. This information would help in the understanding of the correlations and the importance of some physical qualities. Additionally, the sample size was not large, which may reduce the generalization capacity of the evidence found. Moreover, kinematic analysis should be considered for future research aiming to determine the influence of kinematics on the relationships with physical qualities. Despite the limitations, this study is one of the few that exist on the determinants of sprinting performance in adult women soccer players.

## Practical implications

The results revealed possible directions for practical applications. As an example, to sustain stride frequency, lower-limb power should be emphasized in training, namely, using reactive strength (e.g., plyometric training) and improving potentiation training in vertical forces. On the other hand, to improve the sprinting performance (time), the focus should be provided in two different approaches: (1) acceleration and (2) maximal speed. For acceleration, concentric-based training focused on horizontal forces should be emphasized, while for maximal speed, eccentric-based explosive training should be the priority.

## Conclusions

This study revealed that peak power, maximal speed, and lower-limb power (in vertical and horizontal jumps) were significant determinants of sprinting performance (time), while vertical jumps were the determinant of stride frequency. This suggests that potentiation and explosive vertical power could be the emphasis for sustaining the stride frequency of women soccer players, while sprinting performance should be supported by strong acceleration and maximal velocity sustained by both vertical and horizontal forces and concentric and eccentric strength and power.

## Data Availability

The datasets generated during and analyzed during the current study are available from F.T.G.F. on reasonable request.

## Abbreviations

RSA: Repeated-sprint ability
SL: Stride length
SF: Stride frequency
CMJ: Countermovement jump
BIA: Bioelectrical impedance analysis
P_min_: Peak power minimum
P_max_: Peak power maximum
FI: Fatigue index
